# Novel lines of *Pax6*^-/- ^embryonic stem cells exhibit reduced neurogenic capacity without loss of viability

**DOI:** 10.1186/1471-2202-11-26

**Published:** 2010-02-24

**Authors:** Jane C Quinn, Michael Molinek, Tomasz J Nowakowski, John O Mason, David J Price

**Affiliations:** 1 Centre for Integrative Physiology, University of Edinburgh, Hugh Robson Building, George Square, Edinburgh, EH8 9XD, UK; 2 School of Animal and Veterinary Sciences, Charles Sturt University, Boorooma Street, Wagga Wagga, New South Wales 2678, Australia

## Abstract

**Background:**

Embryonic stem (ES) cells can differentiate into all cell types and have been used extensively to study factors affecting neuronal differentiation. ES cells containing mutations in known genes have the potential to provide useful in vitro models for the study of gene function during neuronal differentiation. Recently, mouse ES cell lines lacking the neurogenic transcription factor Pax6 were reported; neurons derived from these *Pax6*^-/- ^ES cells died rapidly after neuronal differentiation in vitro.

**Results:**

Here we report the derivation of new lines of *Pax6*^-/- ^ES cells and the assessment of their ability to survive and differentiate both in vitro and in vivo. Neurons derived from our new *Pax6*^-/- ^lines were viable and continued to elaborate processes in culture under conditions that resulted in the death of neurons derived from previously reported *Pax6*^-/- ^ES cell lines. The new lines of *Pax6*^-/-^ES cells showed reduced neurogenic potential, mimicking the effects of loss of Pax6 in vivo. We used our new lines to generate *Pax6*^-/- ^↔ *Pax6*^+/+ ^chimeras in which the mutant cells survived and displayed the same phenotypes as *Pax6*^-/- ^cells in *Pax6*^-/- ^↔ *Pax6*^+/+ ^chimeras made by embryo aggregation.

**Conclusions:**

We suggest that loss of Pax6 from ES cells reduces their neurogenic capacity but does not necessarily result in the death of derived neurons. We offer these new lines as additional tools for those interested in the generation of chimeras and the analysis of in vitro ES cell models of Pax6 function during neuronal differentiation, embryonic and postnatal development.

## Background

Pax6 is a highly-conserved transcription factor whose main sites of expression are in the developing eye and central nervous system [1-5]. Homozygous loss-of-function mutations of *Pax6 *cause failure of eye morphogenesis and severe abnormalities of brain development [6,7]. Pax6 plays an important role promoting neurogenesis; in vivo, loss of Pax6 results in neural progenitors having reduced neurogenic potential [8,9] whereas its over-expression in vitro pushes cells towards a neuronal fate [8,10,11]. The mechanisms by which Pax6 directly promotes neurogenesis are not yet known.

Pluripotent embryonic stem (ES) cell lines have provided a means to exploit gene targeting for the analysis of gene function *in vivo*. In addition, since ES cell lines can be differentiated into a variety of cell types in culture they provide an opportunity to study gene function by comparing the phenotypes of ES-derived cells *in vitro*. ES cells have been used systematically as a model system for examining factors controlling differentiation pathways in vitro. In particular, differentiation protocols have been reported which recapitulate differentiation of various neuronal cell types, or their precursors, in vitro [12-17].

Recently, ES cell lines derived from mouse embryos lacking Pax6 (*Pax6*^-/-^) were studied in culture [11]. Neurons derived from these *Pax6*^-/- ^ES cells died rapidly after induction of neuronal differentiation, with almost none surviving beyond about a week after plating. This is surprising as *Pax6*^-/-^neurons can survive into postnatal life following transplantation into wild-type embryos [18] and following early embryonic neural tissue-specific conditional deletion of *Pax6 *[19] suggesting that loss of Pax6 alone is not necessarily sufficient to abolish neuronal viability.

We derived new lines of *Pax6*^-/- ^ES cells and assessed their ability to survive and differentiate both in vitro and in vivo. These new lines (which are labelled with a developmentally neutral nuclear transgenic marker) were used to generate *Pax6*^-/- ^↔ *Pax6*^+/+ ^chimeras in which the mutant cells survived and displayed the same phenotype as *Pax6*^-/- ^cells in *Pax6*^-/- ^↔ *Pax6*^+/+ ^chimeras made by embryo aggregation [9,20-22]. In vitro, we found that the new lines of *Pax6*^-/- ^ES cells showed reduced neurogenic potential, mimicking the effects of loss of Pax6 in vivo. Contrary to previous reports that ES-derived cells lacking Pax6 show reduced process development as little as 4 days after plating and die 2-4 days later [11], cells derived from these new *Pax6*^-/- ^lines were viable and continued to elaborate their processes after comparable culture times under comparable conditions. We offer these lines as a validated tool for investigation of gene function during neuronal differentiation in vitro and the generation of chimeric animals for in vivo analysis of development of the central nervous system.

## Methods

### Animals

Animals in this study were bred in-house following Home Office (UK) regulations and were approved by the University of Edinburgh Ethical Review Panel.

### Derivation of *Pax6*^-/- ^ES cells

ES cells that were either wild type (*Pax6*^+/+^) or *Pax6*^*SeyEd*/*SeyEd *^(designated here as *Pax6*^-/-^; [23] were derived using the following protocol. Female mice [129Sv(Ola); *Pax6*^+/-^] were superovulated and mated with males [129Sv(Ola)] that were *Pax6*^+/- ^and homozygous for a reiterated *β-globin *repeat transgene (*Tg/Tg*; [24], which acts as a developmentally neutral marker detectable by DNA-DNA in situ hybridization [9,20,22,25]. Delayed implantation (diapause) was induced at 2.5 days post coitum (dpc) by intraperitoneal injection of Tamoxifen (Sigma, UK; 10 μg/animal) and subcutaneous injection of Depo-Provera (Sigma, UK; 1-3 mg/animal). At 7.5 dpc delayed blastocysts were flushed from the uterus, transferred to a gelatinised well containing N2B27 medium (Stem Cell Sciences, UK) with 10 ng/ml Leukaemia Inhibitory Factor (LIF) and cultured at 37°C in 5% CO_2_. After approximately 5 days in culture, the inner cell mass was detached from the bottom of each well using a fine pulled Pasteur mouth pipette before being disaggregated in trypsin (0.025% for 2-3 minutes at 37°C) and disrupted to give individual clusters of between 1-5 cells. Cell clusters were then transferred to a fresh gelatinised well containing N2B27 media containing LIF (1000 U/ml) with the addition of BMP4 (10 ng/ml). Primary colonies of ES cells were visible after approximately 5 days in culture. ES cell lines were passaged at least twice in feeder-free conditions in BHK-21 Glasgow MEM (GMEM; Sigma, UK) with 15% fetal bovine serum (FBS) and LIF (1000 U/ml). Cell lines were genotyped as described previously [9]. All cell lines were karyotyped to confirm normal chromosome complement.

### Chimera production

Chimeric embryos were produced by blastocyst injection and processed histologically, by in situ hybridization and immunohistochemistry, to detect cells carrying the *Tg *marker and to analyse their expression of transcription factors, as described before [9].

### Neural differentiation

The neural differentiation protocol followed a previous description [14]. Briefly, ES cells maintained in feeder-free conditions were plated in non-adherent dishes in GMEM +15% FBS in the absence of LIF (designated Day 0, or day of LIF withdrawal). After 4 days, all-trans retinoic acid (5 μM, Sigma, UK) was added. The developing embryoid bodies (EBs) were allowed to develop for a further 2 or 4 days (to Days 6 or 8) before dissociation using Tryple Express™ (Invitrogen, UK). EBs taken on Day 6 and some of those taken on Day 8 were exposed to 40 μg/ml bromodeoxyuridine (BrdU) for 30 minutes before being dissociated and plated for two hours in 8-well glass chamber slides, fixed with paraformaldehyde and processed for immunohistochemistry. Other cells dissociated on Day 8 were plated at 2.0 × 10^5 ^cells/cm^2^and cultured in serum-free GMEM/F12:Neurobasal medium (1:1) supplemented with N2 and B27 (Invitrogen, UK) for 6 or 8 days (to Days 14 or 16 after LIF withdrawal) before fixation and processing.

### Primary cortical progenitor cell cultures

The cortices of E11.5 embryos generated from timed matings of *Pax6*^+/- ^animals were isolated. Each cortex was kept separate, its cells were dissociated into separate wells and were allowed to grow for 7 days in DMEM:F12 1:1 culture media supplemented with 100 μg/ml transferrin, 25 μg/ml insulin, 20 nm progesterone, 30 nm sodium selenite, 60 μg/ml putrescine, 20 ng/ml EGF and bFGF (all Sigma, U.K). The genotypes of individual embryos were discovered by PCR analysis as described previously [20]. Primary neurospheres were dissociated on Day 7 and stained with propidium iodide for analysis using flow cytometry.

### Immunohistochemistry and flow cytometry analysis

Antibodies used in this study were against β-III-tubulin (Sigma, UK), microtubule associated protein 2 (MAP2; Sigma), UK, Pax6 (Developmental Studies Hybridoma Bank, USA), glial fibrillary acid protein (GFAP; DAKO, UK) and BrdU (Beckton Dickinson, UK). Visualisation was achieved using Alexafluor-488 or Alexafluor-568 (Invitrogen, UK) conjugated secondary antibodies and cell nuclei were counterstained using TOPRO3 (Molecular Probes, USA). For counting, 3-8 areas were selected randomly per well. Apoptotic cells were identified by their dense chromatin condensation visible with TOPRO3 staining. Chromatin condensation was easily recognized, is a defining feature of apoptosis [26] and has been shown previously to coincide with other indicators of apoptosis such as terminal deoxynucleotidyl transferase-mediated biotinylated UTP nick end labelling (TUNEL) [27].

For flow cytometry, cells were stained with propidium iodide to allow discrimination of single cells and analysis of DNA content. Staining reactions were carried out in duplicate. Cells were analyzed on a Beckman-Coulter XL flow cytometer with Expo32 software. 8,000-20,000 cells were analyzed per sample.

### In vitro transfection

Sub-confluent *Pax6*^-/- ^ES cells were dissociated using Tryple Express™ and plated in GMEM media containing LIF and FBS. Twenty-four hours later they were co-transfected using Fugene (Invitrogen, UK) with pCMV-Script plasmid containing the full length *Pax6 *cDNA [10] and plasmid pEGFPN1 (Clontech, USA) expressing green fluorescent protein (GFP). Transfected cells were selected by culture in medium containing G-418 (500 μg/μl). After 7 days in selection medium individual colonies were picked and expanded by routine passage in GMEM+FBS+LIF. Stably transfected cell lines were subjected to neural induction as described above and cultures fixed for immunohistochemistry at Day 14.

## Results

### Derivation of *Pax6*^-/- ^ES cell lines and generation of *Pax6*^-/- ^↔ *Pax6*^+/+ ^chimeras

We established 9 lines of *Pax6*^+/+ ^ES cells (wtMM1-9) and three lines of *Pax6*^-/- ^ES cells (SeyD1-3) containing the *Tg *transgene [24]. Karyotyping of these ES cell lines showed that each had a normal chromosome complement [28]. *Pax6*^-/- ^ES cell lines displayed no abnormalities of growth during routine passage; two (SeyD1 and SeyD2) were analysed in detail.

**Figure 1:**
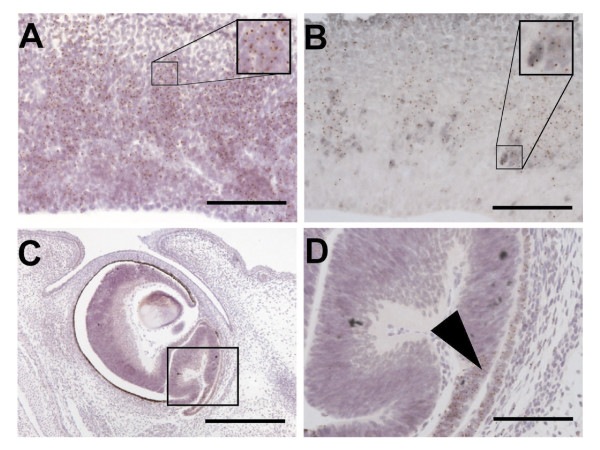
***Pax6*^-/- ^cells derived from mutant ES cells integrate in *Pax6*^-/- ^↔ *Pax6*^+/+ ^chimeras and show predictably abnormal phenotypes**. (A-D) In these E14.5 chimeras, mutant cells are recognized by a brown nuclear spot, which is the result of DNA-DNA in situ hybridization for a reiterated β-globin transgene. (A,B) Sections of the cerebral cortex showing clusters of *Pax6*^-/- ^cells in the subventricular zone; these cells are positive for Mash1 (grey staining in B). (C,D) In the eye, chimeras show abnormal retinal morphologies (boxed area) exhibiting extreme segregation between wild type and mutant cells, similar to abnormalities reported previously in aggregation chimeras [20]. The arrowhead in D indicates an example of the boundary between an area of mutant and an area of wild type cells with abnormal retinal folding. Scale bars: A,B,D = 50 μm; C = 100 μm.

**Figure 2:**
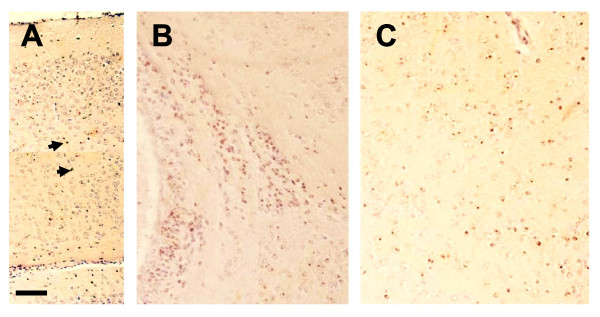
***Pax6*^-/- ^cells derived from mutant ES cells are still present in postnatal *Pax6*^-/- ^↔ *Pax6*^+/+ ^chimeras**. Mutant cells clustered (A) in cortex, (B) at the corticostriatal boundary and (C) in the hippocampus at P5; arrows in A point to examples of labelled mutant cells. Scale bar: A = 50 μm; B,C = 30 μm.

The developmental potential of wild type line wtMM4 and *Pax6*^-/- ^line SeyD1 was assessed by injection into wild type blastocysts to create chimeras. We produced ten *Pax6*^-/- ^↔ *Pax6*^+/+ ^chimeras aged between embryonic day (E) 10.5 and postnatal day (P) 5 and ten control *Pax6*^+/+ ^↔ *Pax6*^+/+ ^chimeras aged between E10.5 and E14.5. In controls, *Tg*+ cells derived from wtMM4 were distributed throughout eye and brain tissues, which exhibited no abnormal phenotypes. In *Pax6*^-/- ^↔ *Pax6*^+/+ ^chimeras, *Tg*+*Pax6*^-/- ^cells derived from SeyD1 showed abnormal distributions in eye and brain tissues, similar to those reported previously for *Pax6*^-/- ^cells in *Pax6*^-/- ^↔ *Pax6*^+/+ ^chimeras produced by aggregation of *Pax6*^+/+ ^and *Pax6*^-/- ^morulae [9,20,22,25]. For example, *Pax6*^-/- ^cells derived from SeyD1 formed clusters in the subventricular zone of the embryonic cortex (Figure 1A, B); these clusters expressed inappropriate markers of ventral telencephalic identity, such as Mash1 (Figure 1B), as predicted from previous work [9,22]. *Pax6*^-/- ^cells derived from SeyD1 failed to contribute to the lens and exhibited extreme segregation from wild type cells in the neural retina, which exhibited areas of characteristic abnormal retinal folding (Figure 1C, D), again as anticipated from previous studies [20]. Clusters of *Pax6*^-/- ^cells derived from SeyD1 were still present in the brains of postnatal chimeras (Figure 2). These findings indicate that cells derived from *Pax6*^-/- ^ES cells can contribute in predictably abnormal ways to the eyes and brains of chimeras for periods extending into postnatal life, long after the onset of neurogenesis.

### Comparison of proliferation, apoptosis and onset of differentiation in wild type and *Pax6*^-/- ^cells derived from wtMM4, SeyD1 and SeyD2 at Days 6-8 in vitro

**Figure 3:**
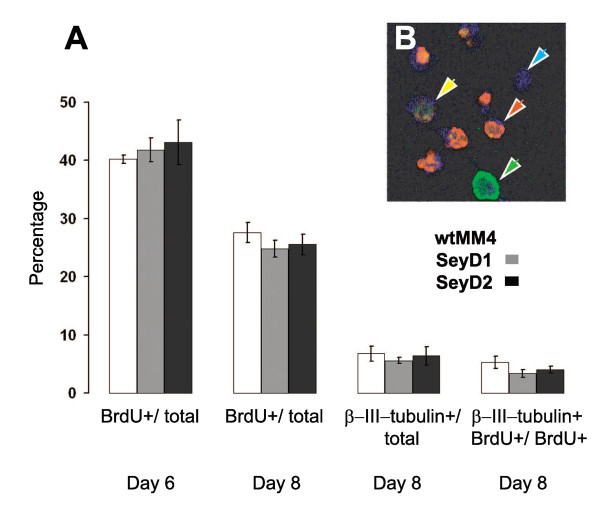
***Pax6*^-/- ^mutant ES cells generate embryoid bodies containing normal proportions of proliferating and differentiating cells**. (A) Histograms show percentages of cells positive for either BrdU, β-III-tubulin or both two hours after plating cells from dissociated EBs that had been exposed to a short pulse of BrdU shortly before dissociation. From left to right: percentages of all cells that were BrdU positive on Day 6 after LIF withdrawal; percentages of all cells that were BrdU positive on Day 8; percentages of all cells that were β-III-tubulin positive on Day 8; percentages of BrdU positive cells that were β-III-tubulin positive on Day 8. All values are means of 3 or 4 independent culture runs ± standard errors. No significant difference was observed between the wild type and *Pax6*^-/- ^mutant ES cell lines within each culture paradigm. (B) Examples of wtMM4 cells that are BrdU positive (red), β-III-tubulin positive (green), double-labeled (yellow arrow) and labeled only with TOPRO3 counterstain (blue).

It is known that many Day 8 cells from wild-type embryoid bodies made with the protocol used here express markers of neural progenitors [11]. In agreement with this, only a few percent of Day 8 cells from embryoid bodies from our new wild-type wtMM4 ES cell line expressed the marker of differentiating neurons, β-III-tubulin (Figure 3). Most of the cells that did not express neuronal markers were positive for the marker of neural progenitors, Nestin (60.7% ± 3.2 s.e.m., n = 4 cultures), and the marker of cortical apical progenitors, RC2 (51.7% ± 3.5 s.e.m., n = 4 cultures).

Embryoid bodies (EBs) from previously-derived *Pax6*^-/- ^ES cells contained normal complements of neuronal progenitors [11]. In agreement with this, we found that proportions of BrdU-labelled proliferating cells were no different between wtMM4, SeyD1 and SeyD2 EBs (Figure 3). We also compared proportions of cells positive for the progenitor markers RC2 and Nestin in EBs on Day 8 and found no evidence of differences between the proportions of progenitors in EBs from our new wild type and *Pax6*^-/- ^lines, in agreement with a similar comparison by Nikoletopoulou et al. [11] of their wild-type and mutant lines.

Proportions of BrdU-labelled proliferating cells declined between Day 6 and Day 8 after LIF withdrawal, coinciding with the onset of differentiation (Figure 3). Small proportions of β-III-tubulin-expressing cells were detected on Day 8 after LIF withdrawal and small proportions of cells labelled with BrdU on Day 8 were also β-III-tubulin-positive several hours later. There were no differences in the proportions of early differentiated neurons between cultures from wild type and *Pax6*^-/- ^lines at Day 8 (Figure 3). Overall, these data indicate that neuronal differentiation is just beginning on Day 8 and the time of onset is not perturbed by the loss of Pax6 expression in our ES cells.

Proportions of apoptotic cells on Day 6 and Day 8 after LIF withdrawal were low with no significant differences between the wild-type and the *Pax6*^-/- ^ES cells (Day 6: *Pax6*^+/+ ^mean percentage of apoptotic cells = 5.42% ± 1.82 s.e.m., n = 5 cultures; *Pax6*^-/- ^mean = 7.64% ± 3.05, n = 5; Day 8: *Pax6*^+/+ ^mean = 2.44% ± 1.27, n = 5; *Pax6*^-/- ^mean = 1.62% ± 0.65, n = 5).

### Reduced proportions of neurons in cells of SeyD1 and SeyD2 lines at Day 14 in vitro

**Figure 4:**
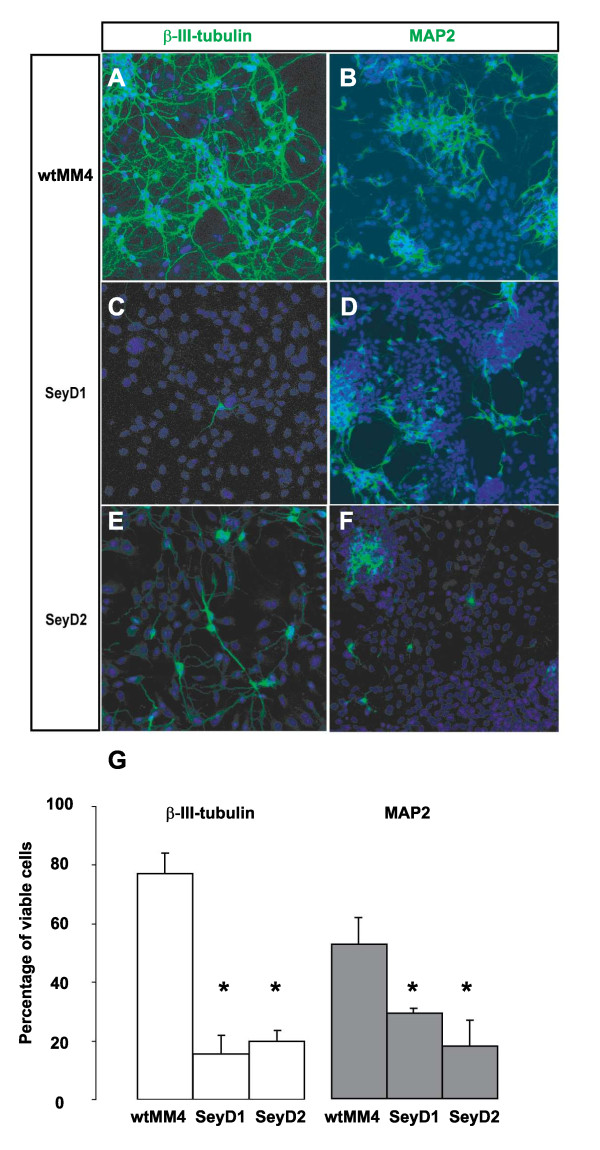
***Pax6*^-/- ^ES cells generate abnormally low proportions of neurons**. (A-F) Examples of cultures of cells derived from wtMM4, SeyD1 and SeyD2 lines on Day 14 after LIF withdrawal stained with either β-III-tubulin or MAP2 (green) and TOPRO-3 (blue). (G) Mean percentages (± standard error of the mean; n = 3-4 independent cultures per line) of β-III-tubulin (open bars) or MAP2 (grey bars) expressing neurons are shown for each ES cell line. Percentages of β-III-tubulin-positive and MAP2-positive neurons were significantly reduced in Pax6 mutant ES cell lines SeyD1 and SeyD2 (with Student's t-tests, differences for β-III-tubulin were both p < 0.0001 and for MAP2 were both p < 0.02).

Previous reports have shown that the differentiation protocol used in this study induces a large majority of β-III-tubulin expressing neurons from wild type ES cells after several days in vitro [14]. Similarly, we found that about 80% of dissociated cells from the wild type ES cell line wtMM4 were positive for β-III-tubulin on Day 14 after LIF withdrawal) with about 50% of cells positive for the later neuronal marker MAP2 (Figure 4A, B, G). In contrast, *Pax6*^-/- ^ES cell lines SeyD1 and SeyD2 lines showed a significantly lower proportion of cells positive for β-III-tubulin and MAP2 (around 20-30%) in Day 14 cultures (Figure 4C-G). These findings indicate that ES-derived progenitors lacking Pax6 have reduced neurogenic potential, as do *Pax6*^-/- ^primary progenitors derived from mutant embryos and *Pax6*^-/- ^cells developing in the cortex of Pax6 mutant chimeric embryos [8,9].

**Figure 5:**
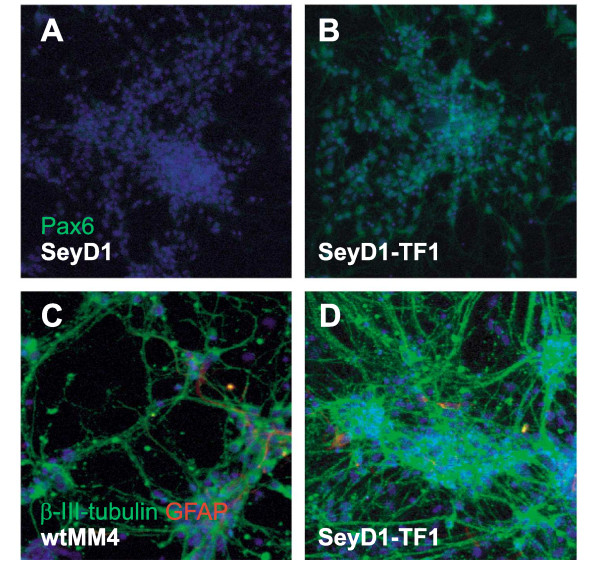
**Re-expression of Pax6 induces extensive neurogenesis from *Pax6*^-/- ^ES cells**. (A, B) Immunocytochemistry for Pax6 (green) does not label cells derived from the founder *Pax6*^-/- ^line SeyD1 (A) whilst Pax6 expression is observed in cells generated from this line that were stably transfected with a Pax6-expression construct (SeyD1-TF1) (B). (C) At Day 14, wild type ES cell-derived cultures contain large numbers of β-III-tubulin-expressing neurons (green; see also Figure 4) with small proportions of GFAP-positive cells (red). (D) At Day 14, almost all SeyD1-TF1 cells (i.e. *Pax6*^-/- ^cells containing a Pax6-expression construct) are β-III-tubulin-expressing neurons (contrasting with results from untransfected *Pax6*^-/- ^cells at this age, see Figure 4), with a small proportion of GFAP-positive cells.

To confirm that the reduced neurogenic potential of our SeyD1 and SeyD2 ES cell lines was due to loss of Pax6, we generated two stably transfected *Pax6*^-/- ^ES cells lines, SeyD1-TF1 and SeyD1-TF2, which expressed full length Pax6 transcript under the control of a constitutive promoter. Pax6 expression in these cells was confirmed by immunohistochemistry to Pax6 protein (Figure 5A, B). SeyD1-TF1 and SeyD1-TF2 ES cell lines generated cultures that, on Day 14, consisted almost entirely of neurons. These neurons produced long processes similar to those seen in wild-type cultures of wtMM4 neurons (Figure 5C, D). Only very small numbers of cells could be identified in each SeyD1-TF1 and SeyD1-TF2 culture which did not express the marker of early neuronal differentiation, β-III-tubulin (Figure 5D). Accurate quantification of the β-III-tubulin-negative cells was not possible due to the extensive process development, but it was clear that proportions of neurons were much higher than the 20-30% observed in the founder mutant SeyD1 and SeyD2 lines (Figure 4G). These results indicate that transfection of SeyD1 and SeyD2 with a construct expressing the full length Pax6 transcript reversed their defective neurogenic potential.

### Continuing development of cells derived from SeyD1 and SeyD2 at Day 16

**Figure 6:**
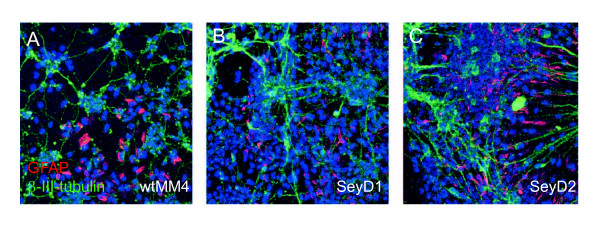
**Neurons from *Pax6*^-/- ^ES cell lines remain viable and continue to elaborate processes after 16 Days in culture**. (A) Wild-type wtMM4 cells and (B) *Pax6*^-/- ^cells from the SeyD1 and (C) SeyD2 lines labeled for β-III-tubulin (green) and GFAP (red), with TOPRO-3 counterstaining (blue). Cells derived from all three ES cell lines are viable and have continued to make elaborate processes.

To determine cell viability after longer periods in culture, we allowed cells derived from our *Pax6*^+/+^, *Pax6*^-/- ^SeyD1 and SeyD2 lines to continue to grow in culture to Day 16 post LIF withdrawal. At this time under comparable culture conditions, neurons derived from previously reported *Pax6*^-/- ^ES cell lines had all died [11]. In contrast, neurons derived from our *Pax6*^-/- ^SeyD1 and D2 lines continued to elaborate large meshworks of β-III-tubulin-positive processes (Figure 6) showing no noticeable loss of viability after 16 days in culture.

As at Day 14, many *Pax6*^-/- ^SeyD1 and D2 cells remained negative for β-III-tubulin at Day 16 but the extensive processes surrounding all cells in the cultures made it impossible to count proportions of these β-III-tubulin-negative cells accurately. We found that some cells in wtMM4 and SeyD1 and SeyD2 lines were positive for GFAP, a marker of cells of glial identity (Figure 6).

### Primary neural progenitors lacking Pax6 do not exhibit enhanced cell death

To test whether primary neural progenitors derived from *Pax6*^-/- ^embryos showed increased cell death in culture, neural progenitors were isolated from wild type and Pax6 mutant cortex at E11.5 and expanded in vitro for 7 days. Neurospheres were dissociated and proportions of dead cells were determined from the size of the hypodiploid (subG1) population of cells by flow cytometry as described previously [8]. No significant difference was observed in the proportions of hypodiploid cells between the wild-type and mutant cells (mean percentages of cells in subG1 were: *Pax6*^+/+^, 12.6% ± 2.10 s.e.m.; *Pax6*^-/-^, 7.8% ± 2.85, n = 3 cultures each). These data indicate that loss of Pax6 in primary neural progenitors does not increase cell death.

## Discussion

In many respects, the in vitro development of our new *Pax6*^-/- ^ES cells resembles that of previously derived *Pax6*^-/- ^ES cells [11] and that of primary *Pax6*^-/- ^progenitors taken from mutant embryos [8]. Firstly, our new wild-type and *Pax6*^-/- ^mutant lines produced EBs containing proliferating progenitors in large proportions that were similar to those in EBs of wild type ES cells and of previously derived *Pax6*^-/- ^ES cells. This agrees with a previous conclusion that the generation of early neurogenic progenitors from ES cells is not perturbed in the absence of Pax6 [11]. Secondly, Nikoletopoulou et al. [11] showed that abnormally high proportions of these progenitors expressed the ventral telencephalic marker Mash1; similarly, we found abnormal ectopic expression of Mash1 in *Pax6*^-/- ^ES-derived cells in the dorsal telencephalon of chimeras. Thirdly, previous studies have shown that primary *Pax6*^-/- ^progenitors from mutant cortex generate reduced proportions of neurons in culture [8]; we found the same trend in cells differentiated from our lines of *Pax6*^-/- ^ES cells in vitro. Together these data suggest that our novel *Pax6*^-/- ^ES cell lines are mimicking the in vivo and in vitro characteristics of neuronal progenitors derived from primary tissue of the *Pax6*^-/- ^embryo.

In one respect, however, our new *Pax6*^-/- ^ES cell lines developed quite differently to *Pax6*^-/- ^ES cell lines reported previously. Our *Pax6*^-/- ^ES cell-derived neurons showed no signs of early failure of process development and the extensive cell death previously reported; on the contrary, they were continuing to develop ever more elaborate processes at an age when virtually all of the previously described cells had died [11].

Since the culture protocol we used is similar to that used to differentiate previously derived *Pax6*^-/- ^ES cells, possible explanations for this difference in viability must centre of the nature of the ES cells themselves. One possibility is variation in genetic background. Our ES cells were derived from Pax6 mutant mice inbred on a 129Sv(Ola) background whereas those used by Nikoletopoulou et al. [11] were not (N.D. Allen, personal communication). It is possible that genetic background is critical for the manifestation of abnormally high levels of cell death observed in the previous study. This may not be surprising as genetic background has been shown to play a role in determining the exact nature of the eye and craniofacial defects associated with the *Pax6*^-/- ^phenotype [29].

Nikoletopoulou et al. [11] did not report the phenotype of *Pax6*^-/- ^cells derived from their mutant ES cells in mouse chimeras. They did, however, describe the introduction of *Pax6*^-/- ^ES cell derived progenitors into chick telencephalon: some of the neurons derived from these cells survived in the chick telencephalon, suggesting that even in these cells the absence of Pax6 does not necessarily cause extensive cell death given an appropriate supporting environment. Therefore induction of Pax6 genotype-dependent neuronal cell death might depend on both the genetic background of the mutant cells and the environment in which they are placed. It seems quite conceivable that loss of Pax6 from ES cells might cause the rapid death of all derived cells with some genetic backgrounds in vitro but not in vivo, whereas loss of Pax6 from ES cells with other genetic backgrounds might generate cells that are viable both in vitro and in vivo, as is the case in our novel Pax6 mutant cell lines.

Although Nikoletopoulou et al. [11] suggested that the rapid death of all *Pax6*^-/- ^ES cell derived neurons in culture reflects an increased incidence of cell death in the cerebral cortex of *Pax6*^-/- ^mice, other studies did not report an increase in apoptotic cells in the *Pax6*^-/- ^cortex in vivo or among *Pax6*^-/- ^primary cells in culture [8,9,30]. Data included in this present study on cultured cells from embryos support previous evidence against an increase in cell death in the absence of Pax6. On the contrary, *Pax6*^-/- ^neurons have been shown to survive for extended periods, even into postnatal life following transplantation into wild type embryos [18] and following early embryonic neural tissue-specific deletion of *Pax6 *[19]. In the present study *Pax6*^-/- ^ES-derived neurons in chimeras also survived into postnatal life. Overall, most published studies point to the importance of Pax6 in the early regulation of neurogenesis [8,9,30-33], whereas the evidence that Pax6 has a prominent role in cell death remains controversial.

## Conclusion

In conclusion, we have successfully derived *Pax6*^-/- ^ES cells that develop in ways predicted from previous work on *Pax6*^-/- ^progenitors and neurons in vivo and in vitro. Neurons derived from our new lines show enhanced viability compared to neurons derived from previously studied *Pax6*^-/- ^ES cell lines. Our new lines offer a good model for the study of *Pax6 *gene function in vivo and in vitro.

## Authors' contributions

JQ and MM carried out the ES cell derivation and JQ, MM and TN performed the differentiation analysis. JQ, JM and DP conceived of the study, participated in its design and coordination and helped to draft the manuscript. All authors read and approved the final manuscript.
